# Akt-mediated phosphorylation of Oct4 is associated with the proliferation of stem-like cancer cells

**DOI:** 10.3892/or.2015.3752

**Published:** 2015-01-27

**Authors:** QING-WEI ZHAO, YAN-WEN ZHOU, WEN-XIN LI, BO KANG, XIAO-QIAN ZHANG, YING YANG, JIE CHENG, SHENG-YONG YIN, YING TONG, JIAN-QIN HE, HANG-PING YAO, MIN ZHENG, YING-JIE WANG

**Affiliations:** 1 State Key Laboratory for Diagnosis and Treatment of Infectious Diseases, The First Affiliated Hospital, School of Medicine, Zhejiang University, Hangzhou, Zhejiang, P.R. China; 2 Department of Pharmacy, The First Affiliated Hospital, School of Medicine, Zhejiang University, Hangzhou, Zhejiang, P.R. China; 3 Collaborative Innovation Center for Diagnosis and Treatment of Infectious Diseases, Zhejiang University, Hangzhou, Zhejiang, P.R. China; 4 College of Life Sciences, Zhejiang University, Hangzhou, Zhejiang, P.R. China; 5 Department of Hepatobiliary and Pancreatic Surgery, The First Affiliated Hospital, School of Medicine, Zhejiang University, Hangzhou, Zhejiang, P.R. China; 6 Department of Neurosurgery, The First Affiliated Hospital, School of Medicine, Zhejiang University, Hangzhou, Zhejiang, P.R. China

**Keywords:** Akt, Oct4, phosphorylation, proliferation, stem-like cancer cells, glioblastoma

## Abstract

Oct4 protein encoded by *POU5F1* plays a pivotal role in maintaining the self-renewal of pluripotent stem cells; however, its presence in cancer cells remains controversial. In the present study, we provided evidence that the transcripts of authentic OCT4 gene (*OCT4A*) and its multiple pseudogenes were detected in a variety of cancer cell lines. A few major bands were also detected by western blotting using an anti-Oct4A monoclonal antibody. Moreover, an anti-Oct4-pT235 antibody was used to identify a band in the majority of the tested cancer cell lines that coincided with one of the anti-Oct4A bands which was decreasable by a specific shRNA. The Oct4-pT235 signals were also detected in human glioblastoma and liver cancer specimens by immunofluorescence microscopy and immunohistochemistry. U87 glioblastoma cells were cultured in a neural stem cell medium to induce the formation of neurospheres rich in stem-like cancer cells. The levels of Oct4-pT235 in the sphere cells were markedly increased compared to their monolayer parental cells, a result that was accompanied by upregulation of the PI3K-Akt pathway. Akti-1/2, a specific inhibitor of Akt, effectively reduced the level of Oct4-pT235 and attenuated the proliferation of U87 sphere cells. ITE, an agonist of the aryl hydrocarbon receptor, also significantly attenuated the Akt-mediated phosphorylation of Oct4 in glioblastoma and liver cancer cells, and reduced their tumorigenic potential in a xenograft tumor model. Taken together, we concluded that the Akt-mediated phosphorylation of Oct4A or its homolog protein was associated with the proliferation of stem-like cancer cells that may serve as a novel biomarker and drug target for certain types of cancer.

## Introduction

The human *POU5F1* gene, located on chromosome 6p21.3, can generate at least three transcripts (OCT4A, OCT4B and OCT4B1 mRNA) and four protein isoforms (Oct4A, Oct4B-265, Oct4B-190, and Oct4B-164) through alternative splicing and alternative translation initiation (1). Oct4A was found to be expressed in unfertilized oocytes, early embryos, primordial germ cells and different embryonic stem cell (ESC) and embryonal carcinoma cell (ECC) lines (2). Oct4A has been established as a master regulator in the maintenance of self-renewal and pluripotency of ESCs and ECCs (2–4) and is an indispensable transcription factor in the generation of induced pluripotent stem cells (iPSCs) (5–7). Oct4B and Oct4B1 isoforms (including Oct4B-265, 190 and 164), differ from Oct4A in the N-transactivation domain but share identical POU and C-transactivation domains with Oct4A. The Oct4B and Oct4B1 isoforms are considered unable to sustain ESC self-renewal but may play a role in the response to cell stresses (1,2,8). Apart from multiple transcriptional and translational products of *POU5F1*, six *POU5F1* pseudogenes, including processed and non-processed types, that produce transcripts highly homologous to OCT4A mRNA have been reported (9). Some of these pseudogenes detected in certain cancer cell lines and cancer tissues are involved in the regulation of *POU5F1* gene activity and are correlated with poor prognosis of human cancer (10–12). Notably, *POU5F1P1* (also known as *POU5F1B, OCT4-pg1, OTF3C* or *OTF3P1*), a processed pseudogene located on human chromosome 8q24.21 and the best documented pseudogene of *POU5F1*, may encode a protein exhibiting 95% homology with the amino acid sequence of Oct4A (13). Of note, Breyer *et al* (14) found that *OCT4-pg1* expression was positively correlated with *POU5F1* expression in normal prostate tissue and prostate tumors, indicating a coordinative regulation between the two genes.

However, whether *POU5F1* is expressed and Oct4A protein is present in normal and cancer adult tissues and cells remains highly controversial, mainly because the RT-PCR primers and anti-Oct4 antibodies employed did not distinguish Oct4A from other Oct4 isoforms and its pseudogene products (15–19). By employing OCT4A-specific primers and OCT4A-specific restriction digestion of PCR fragments, and by confirming the PCR products with DNA sequencing, Jez *et al* (20) found that OCT4A transcription was undetectable in normal adult human dermal fibroblasts but was significantly induced when the cells were treated with hypoxia and FGF2, leading to a so-called ‘regeneration-competent’ state. Thus, it is possible that *OCT4A* gene in differentiated cells may be re-activated under certain conditions.

In addition to the diversity of the transcription and translation processes of *POU5F1* gene, Oct4 protein was also subjected to multiple post-translational modifications (PTMs) such as sumoylation, ubiquitination and phosphorylation, all of which critically regulate its functions (21–24). Recently, we (25) and other authors (26,27) reported that human Oct4A can be phosphorylated directly by Akt at threonine 235 (T235, equivalent to mouse T228). This site-specific phosphorylation resulted in the stabilization of Oct4A, and the levels of phosphorylated Oct4A (Oct4-pT235) correlated with the resistance to apoptosis and the tumorigenic potential of ECCs (25). As an extension of that study, we examined the expression of Oct4 and Oct4-pT235 in human somatic cancer cell lines and tissues using combinatory approaches. The results suggested that Oct4A or its homolog and Akt-phosphorylated Oct4-pT235 are present in human cancer cells, and that the Akt-Oct4 regulatory circuit was enhanced in neurosphere cells, thereby promoting the self-renewal and survival of these stem-like cancer cells.

## Materials and methods

### Cell lines and culture

293T, NCCIT, U87, SW837, MCF-7 and HepG2 cells were obtained from the American Type Culture Collection (ATCC, Rockville, MD, USA). U251 cells were obtained from Shanghai Bogoo Biotechnology, Co., Ltd. (Shanghai, China). HCCLM3 cells were purchased from the Cell Bank of the Chinese Academic of Sciences, (Shanghai, China). L3.6pl cells, derived from human pancreatic carcinoma (28), were a gift from Professor M.H. Wang (Cancer Biology Research Center, School of Pharmacy, Texas Technical University Health Sciences Center, Amarillo, TX, USA). Most cells were cultured in DMEM (21063-029, Invitrogen, Carlsbad, CA, USA), SW837 cells were maintained in RPMI-1640 medium (11835-030, Invitrogen) and L3.6pl cells were cultured in MEM (51200-038, Invitrogen), all supplemented with 10% heat-inactivated fetal bovine serum (FBS) (10099, Gibco, Carlsbad, CA, USA) and 1% (v/v) penicillin/streptomycin (PS) (15140-148, Gibco). The cells were cultured at 37°C in a humidified 5% CO_2_ incubator (3111, Thermo Fisher Scientific, Waltham, MA, USA).

### Reagents and antibodies

B-27 supplement minus Vitamin A (12587-010) and basic fibroblast growth factor (bFGF) (PHG0266) were obtained from Gibco. Epidermal growth factor (EGF) (E5036), leukemia inhibitory factor (LIF) (L5283) and DMSO (D5879) were purchased from Sigma-Aldrich (St. Louis, MO, USA). The anti-CK19 (ab52625) was obtained from Abcam (MA, Cambridge, USA). ITE was chemically synthesized by KNC Laboratories Co., Ltd. (Tokyo, Japan) (29). The sources of other reagents were previously described (25).

### RT-PCR

Total cell RNAs were extracted using TRIzol reagent (15596-026, Life Technologies, Carlsbad, CA, USA) and the reverse transcription reaction was performed using a PrimeScript RT reagent kit with gDNA eraser (RR047A, Takara, Mountain View, CA, USA). The ‘Total OCT4’ transcripts were amplified by polymerase chain reaction (PCR) in a C1000 Thermal Cycler (Bio-Rad, Hercules, CA, USA) using the primers: forward: 5′-GTGGAGGAAGCTGACAACAA-3′ and reverse: 5′-ATTCTCCAGGTTGCCTCTCA-3′, and the amplified fragment was 120 bp in length. The thermal profile was as follows: 94°C for 2 min; 35 cycles with denaturation at 95°C for 50 sec, annealing at 63°C for 30 sec, and extension at 72°C for 30 sec; with a final extension at 72°C for 5 min. The ‘OCT4A’ transcripts were amplified using the primers (30): forward: 5′-CTTCTCGCCCCCTCCAGGT-3′ and reverse: 5′-AAATAG AACCCCCAGGGTGAGC-3′. The amplified fragment was 496 bp in length, and the thermal profile was as follows: Denaturation at 94°C for 30 sec, annealing at 64°C for 30 sec, and extension at 72°C for 35 sec for 35 cycles, with a final extension at 72°C for 10 min. The PCR products were then subjected to 2% agarose gel electrophoresis with ethidium bromide (15585011, Life Technologies).

### DNA sequencing

A primer pair (forward: 5′-CGGGACA CCTGGCTTCGGAT-3′ and reverse: 5′-CTCAGGCTGAGA GGTCTCCA-3′; with an amplified fragment of 284/285 bp in length) designated as ‘OCT4A+pg134 primers’, targeting the transcripts of OCT4A, OCT4-pg1, OCT4-pg3 and OCT4-pg4, was designed to investigate the transcription and proportion of OCT4A and its major pseudogenes in human cancer cells. The primers were synthesized by Sangon Biotech Shanghai Co., Ltd. (Shanghai, China). The thermal profile used was: 94°C for 3 min; 35 cycles with denaturation at 94°C for 30 sec, annealing at 66°C for 30 sec, and extension at 72°C for 45 sec; at the end of 35 cycles an additional extension step of 5 min at 72°C was added. The PCR products were separated on a 2% agarose gel by electrophoresis and the positive band was excised and extracted from the gel using the SanPrep Column DNA Gel Extraction kit (SK8132, Sangon Biotech Shanghai Co., Ltd.) and then cloned to pEASY-T1 Simple Cloning Vector (CT111, Beijing TransGen Biotech Co., Ltd., Beijing, China). The recombinants were then transfected into DH5α-competent cells (CD201, Beijing TransGen Biotech Co., Ltd.). Ampicillin-resistant clones were selected for sequencing by Sangon Biotech Shanghai Co., Ltd. The obtained DNA sequences were analyzed with the Nucleotide BLAST program and the Lasergene software package (DNA Star Inc., Madison, WI, USA).

### Western blot analysis

Cultured cells with or without treatment were lysed, and the whole cell lysates were separated by SDS-PAGE, blotted onto nitrocellulose membranes, and probed with the indicated antibodies, as previously described (25). GAPDH was used as an internal control.

### Lentiviral vector construction, viral production and viral infection

Lentiviral vector construction and viral production were carried out as described previously (25). For viral infection, U87 cells were plated and cultured overnight, and the culture media were replaced with viral supernatants supplemented with polybrene (AL-118, Sigma-Aldrich) at a final concentration of 8 μg/ml. In most cases, the multiplicity of infection (MOI) was estimated to be between 0.5 and 2. After 8–10 h, the viral supernatants were replaced with fresh culture medium to allow further growth until use.

### Immunofluorescence microscopy and immunohistochemistry

Immunofluorescence microscopy was performed as previously described (25). For immunohistochemistry, surgically resected specimens were collected from patients with liver cancer or glioma prior to therapeutic treatment. Non-cancerous liver or brain tissue specimens were obtained from individuals who had died in accidents. Tissue collection or autopsies were conducted at the First Affiliated Hospital, School of Medicine, Zhejiang University. Written informed consent was obtained from the patients or their relatives and the study was conducted with the approval of the Ethics Committee of the First Affiliated Hospital, School of Medicine, Zhejiang University. Immunohistochemical staining was carried out with paraffin-embedded specimens. Briefly, the sections were cut, de-waxed in 100% xylene twice, rehydrated in a graded alcohol series (100, 95, 80 and 70%, for 5 min each), rinsed with water three times, and incubated in 0.3% H_2_O_2_ in methanol for 20 min to inactivate endogenous peroxidase activity. The sections were then boiled in a microwave oven in 0.01 M citrate buffer solution for 10 min. Each section was incubated with 3% bovine serum albumin (BSA, 9048-46-8, Sigma-Aldrich) for 30 min, followed by incubation with the rabbit anti-Oct4-pT235 antibody (25) diluted in 1% BSA (1:500) overnight at 4°C. The sections were washed with PBS three times, for 5 min each. After incubation with peroxidase-conjugated anti-rabbit secondary antibody (7074, Cell Signaling Technology, Beverly, MA, USA) for 60 min, each section was washed with PBS for three 5-min washes as described above. The sections were visualized by an Olympus IX81 microscope with an Olympus IX-TVAD camera following development in 3,3′-diaminobenzidine tetrahydrochloride (DAB) solution and counterstaining by hematoxylin for 1 min and sealed by neutral gum.

### Mouse xenograft tumor model and immunohistochemistry

BALB/c nude mice were purchased from the Shanghai Experimental Animal Centre, Chinese Academy of Science and maintained as previously described (25). Animal experiments were conducted in accordance with the Guide for the Care and Use of Animals for Research Purposes, and were approved by the Committee of Animal Ethics, Zhejiang University.

The HCCLM3 cell orthotopic xenograft model was established as previously described (31). ITE treatment was performed two weeks after implantation. Each treatment group consisted of three tumor-bearing mice. Vehicle (DMSO) or ITE in the vehicle at 80 mg/kg body weight was administered to the mice by intraperitoneal injection once daily for 15 consecutive days. The mice were then sacrificed. Following tumor excision from the euthanized mice, a portion of the tumor tissue was fixed in 10% formalin for subsequent histological examination as previously described (31). Immunohistochemistry was performed as described above using anti-CK19 (diluted at 1:250) and anti-Oct4-pT235 (diluted at 1:200) antibodies.

### Neurosphere and WST-1 assays

The neurosphere assay was performed as previously described (32). Typical neurospheres formed after cells were cultured in NSC medium for 5 days. Cell proliferation was determined by the WST-1 Cell Proliferation kit (05015944001, Roche Diagnostics, Indianapolis, IN, USA) according to the manufacturer’s instructions.

### DNA microarray

The service for DNA microarray analysis was provided by the Shanghai Biotechnology Corp. (Shanghai, China). Briefly, RNA was extracted from U87 parental and neurosphere cells using the TRIzol method according to the manufacturer’s instructions. Reverse transcription to the first-strand cDNA was primed with T7 oligo(dT) primer to synthesize cDNA containing a T7 promoter sequence. The second-strand cDNA synthesis converted the single-stranded cDNA into a double-stranded DNA (dsDNA) template for transcription. Hybridization was carried out by the GeneChip Hybridization, Wash, and Stain kit (affymetrix: 900720). The arrays were scanned at 570 nm with a confocal scanner from Affymetrix. Analysis of the arrays was performed using the Partek GS 6.5. Normalization of the array was performed using a robust multiarray analysis (RMA). A p-value cut off of 0.05 was used to filter genes that were significantly expressed between the two samples. A fold change of >1.5 was used as a criterion for differential gene expression. Histograms of upregulated genes were prepared using GraphPad Prism 5.0 software (GraphPad Software, Inc., La Jolla, CA, USA).

### Statistical analysis

Statistical analyses were carried out using the SPSS 19.0 statistical software package (IBM Corp., New York, NY, USA). Quantitative data were presented as means ± SD of three independent experiments. The statistical significance was evaluated using the two-tailed unpaired Student’s t-test. P<0.05 was considered statistically significant.

## Results

### Oct4 and Akt-phosphorylated Oct4-pT235 are detected in human cancer cell lines

**Figure 1:**
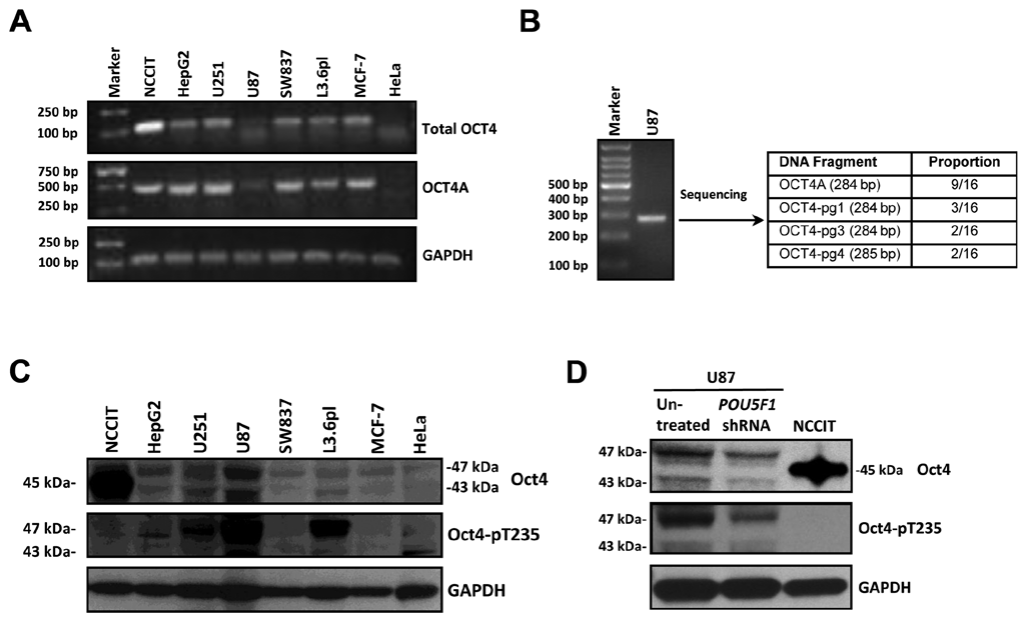
Oct4 and Akt-phosphorylated Oct4-pT235 are expressed in human cancer cell lines. (A) Total RNAs of a number of cultured cancer cell lines were extracted, reverse-transcribed and PCR-amplified using ‘Total OCT4’ primers (top) or ‘OCT4A’ primers (middle), and GAPDH primers (bottom) as a loading control. (B) The OCT4A, OCT4pg-1, OCT4pg-3 and OCT4pg-4 DNA fragments were PCR amplified simultaneously from total RNAs of U87 cells using the ‘OCT4A+pg134 primers’ (left panel) and the positive band (284/285 bp) was excised and extracted from the gel for sequencing as described in Materials and methods. The proportion of each OCT4 fragment based on the sequencing result was presented (right panel). (C) The whole cell lysates of a number of cultured human cancer cell lines (the human NCCIT ECC line was used as a control) were subjected to western blot analysis and the expression of Oct4 (top panel) and Oct-pT235 (middle panel) were detected using the indicated antibodies. GAPDH (bottom panel) served as the loading control. (D) U87 cells were infected with lentiviruses harboring *POU5F1* shRNA for 8 h and then cultured in fresh media for an additional 72 h. Uninfected parental U87 cells served as the blank control. Cells were harvested and lysed, and the whole cell lysates were subjected to western blot analysis using anti-Oct4 (top panel) or anti-Oct-pT235 (middle panel). GAPDH (bottom panel) served as the loading control.

Using a pair of primers that can amplify all known OCT4 isoforms and *POU5F1* pseudogenes (designated as ‘Total OCT4’) (25), we detected the mixed OCT4 transcripts in a variety of human cancer cell lines by RT-PCR (Fig. 1A). The same set of samples was amplified by the OCT4A-specific primers that presumably exclude major known *POU5F1* pseudogenes as described by Atlasi *et al* (30). A clear band with anticipated size (496 bp) was detected in all the tested cancer cell lines with the exception of HeLa cells (Fig. 1A). Furthermore, a single band was visible when the U87 cell sample was amplified with the primers that target the transcripts of OCT4A and three pseudogenes (OCT4-pg1, OCT4-pg3, OCT4-pg4), and DNA sequencing confirmed the presence of all four types of transcripts in U87 cells (Fig. 1B). The anti-Oct4A antibody which identified a strong band of 45 kDa in NCCIT cells detected two major bands in most human cancer cell lines that ran at 47 and 43 kDa, respectively, with the 47 kDa band being stronger in most cases (Fig. 1C). When the electrophoresis conditions were set to optimal, the major 47 kDa band was further resolved into two bands. Using an antibody that can specifically recognize Akt-phosphorylated Oct4 (Oct4-pT235), we detected strong bands in several but not all cell lines that coincided with the 47 kDa band(s) (Fig. 1C). Since U87 cells exhibited the strongest signals for the 47 kDa Oct4 band(s) and the Oct4-pT235 band, we conducted most of the subsequent studies with this cell line. To determine whether any of the above detected protein bands were truly derived from Oct4, we compared the bands in U87 cells treated with or without a *POU5F1* shRNA (25) that can target the 3′UTR of *POU5F1* (including OCT4A, OCT4B and OCT4B1) and *OCT4-pg1*. Of note, both the 47 and 43 kDa bands recognized by either anti-Oct4 or anti-Oct4-pT235 were significantly reduced by the *POU5F1* shRNA, while other non-specific bands remained unchanged (Fig. 1D). Since the reduction of the 47 kDa band by *POU5F1* shRNA was more obviously revealed by anti-Oct4-pT235, it is likely to be the main Oct4 variant that is phosphorylated by Akt in U87 cells. Taken together, our data suggested that Oct4A or its homolog protein (most likely Oct4-pg1) was present in certain human cancer cell lines such as glioblastoma cells and it is phosphorylated by Akt at T235.

### Akt-phosphorylated Oct4-pT235 is predominantly localized in the nucleus of cancer cells

To determine the intracellular localization of Oct4 in human cancer cells, we examined Oct4 and pOct-T235 in the U87 and U251 glioblastoma cell lines by immunofluorescence microscopy, using the anti-Oct4 and anti-pOct-T235 employed in the above western blot analysis and in our previous study (25), with 293T and NCCIT cells being the negative and positive controls, respectively. The nuclei were counterstained with Hoechst 33342. As expected, there was no discernable Oct4 or Oct4-pT235 immunofluorescent signal in 293T cells.

**Figure 2:**
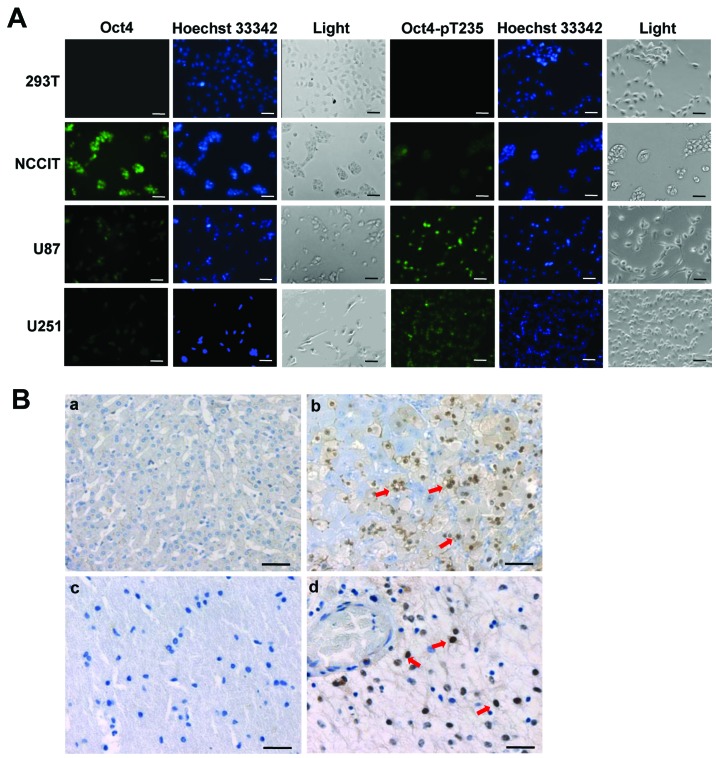
Oct4-pT235 is predominantly localized in the nucleus of cancer cells. (A) U87 and U251 cells were immunostained with anti-Oct4 (green) or anti-Oct4-pT235 (green), respectively, and counterstained with Hoechst 33342 (blue). 293T and NCCIT cells were used as the negative and positive controls, respectively. For all panels, original magnification, ×200; scale bar, 50 μm. (B) Human cancerous and non-cancerous tissues were subjected to immunohistochemical staining with anti-Oct4-pT235 (DAB staining and hematoxylin counterstaining). (a and b) Oct4-pT235 staining of human non-cancerous and cancerous liver sections, respectively. Original magnification, ×200; scale bar, 50 μm. (c and d) Oct4-pT235 staining of human non-cancerous brain tissue and glioma sections, respectively. Original magnification, ×200; scale bar, 50 μm.

Consistent with results of previous studies (25), Oct4 was predominantly localized in the nuclei of NCCIT cells and the immunofluorescent signals were relatively strong (Fig. 2A). By contrast, the Oct4 signals in U87 and U251 cells were much weaker but still discernable. Notably, the immunofluorescence intensity of Oct4-pT235 exhibited an opposite pattern where the signals seen in the two glioblastoma cell lines were much stronger than those in NCCIT cells although all the signals were mainly detected in the nucleus (Fig. 2A). To further confirm the presence and the subcellular localization of Oct4-pT235 in human cancer cells, we then conducted immunohistochemical analysis of Oct4-pT235 in tissue specimens from normal and liver cancer or glioblastoma patients. The results showed that Oct4-pT235 was present at certain regions in the human liver cancer and glioblastoma specimens but rarely in normal liver and brain specimens (Fig. 2B), and consistent with the immunofluorescence data, Oct4-pT235 was predominantly localized in the nucleus of cells in those cancer tissues. Collectively, our data indicated that Oct4A or its homolog protein is present in the nucleus of certain human cancer cells and a significant portion of this Oct4 is phosphorylated at T235 by Akt.

### Level of Akt-phosphorylated Oct4-pT235 is increased in glioblastoma stem-like cells

It has been established that when glioblastoma cells such as U87 cells are cultured in a neural stem cell (NSC) medium to induce the formation of neurospheres, a number of stem cell markers are upregulated and therefore such neurospheres are considered to be rich in glioblastoma stem-like cells (32,33).

**Figure 3:**
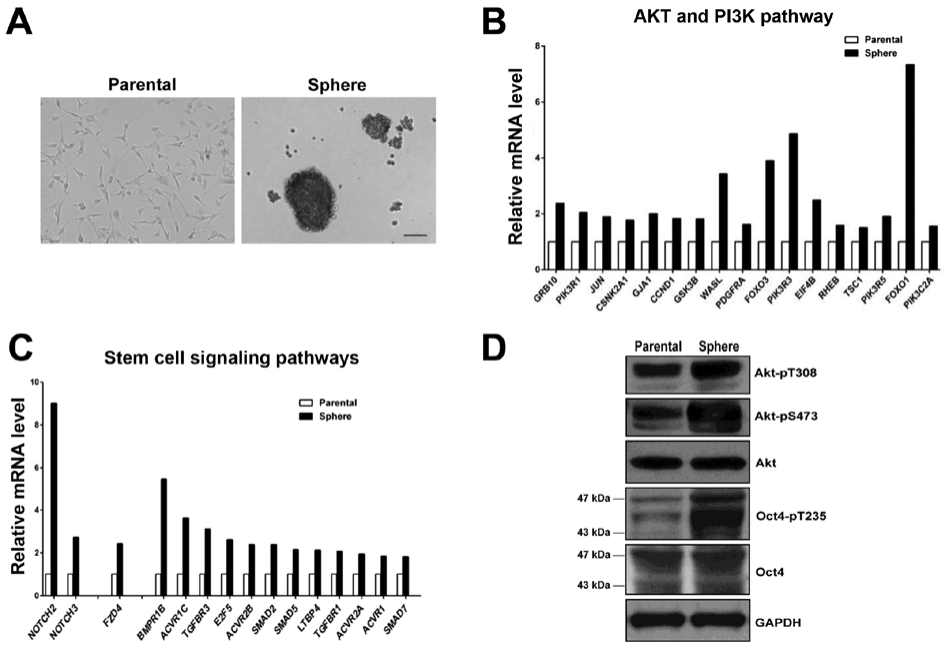
Increased PI3K-Akt-Oct4 pathway in glioblastoma stem-like cells. (A) Morphology of U87 cells cultured in routine DMEM medium (Parental) and NSC medium (Sphere), respectively. Scale bar, 100 μm. (B and C) Total RNAs of U87 parental cells and U87 neurosphere cells were extracted and subjected to genome-wide DNA microarray analysis. Relative mRNA levels of genes involved in (B) PI3K-Akt pathway and (C) several key stem cell signaling pathways were compared between the two groups. The values of U87 parental cells (white bars) were normalized to 1. (D) The whole cell lysates of U87 parental and U87 neurosphere cells were subjected to western blot analysis with the indicated antibodies. GAPDH served as the loading control.

We obtained the U87 neurospheres that exhibited the identical morphological features as those reported in the literature (Fig. 3A). A genome-wide DNA microarray analysis was conducted to comprehensively compare the transcriptional profiles between U87 parental and sphere cells. A total of 4,642 genes were found to be upregulated in U87 neurosphere cells. Among them, some were associated with the PI3K-Akt pathway (Fig. 3B), and a number of stem-cell related pathways such as the Notch, Wnt and TGFβ pathways (Fig. 3C). We then compared the protein levels of Akt and Oct4 between U87 parental cells and corresponding neurosphere cells by western blot analysis. While there was no discernable change in the total Akt level in U87 neurosphere cells, the levels of Akt-pT308 and particularly Akt-pS473 were markedly enhanced. Similarly, although the total Oct4 level was similar between U87 parental and sphere cells, the Oct4-pT235 level in sphere cells was significantly higher than that in parental cells (Fig. 3D). Collectively, our data showed that the PI3K-Akt signaling pathway and the major stem cell self-renewal pathways were upregulated in glioblastoma stem-like cells, and this was associated with an increased Oct4-pT235 level in those cells.

### Inhibition of Akt activity decreases the Oct4-pT235 level and attenuates the proliferation of glioblastoma stem-like cells

**Figure 4:**
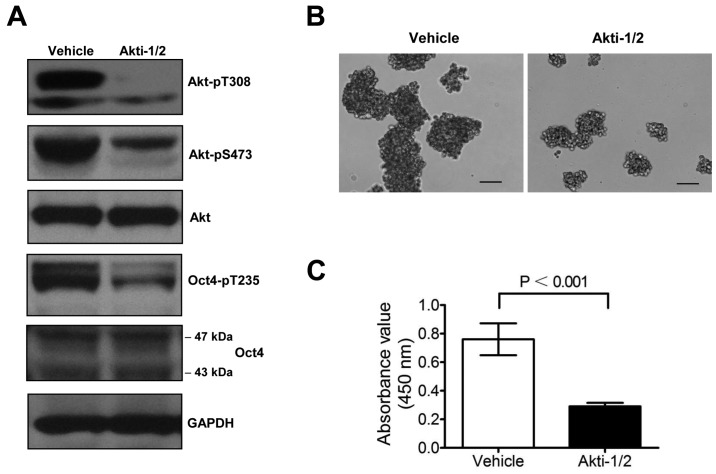
Inhibition of Akt activity by Akti-1/2 decreases the Oct4-pT235 level and attenuates the proliferation of glioblastoma stem-like cells. (A) U87 cells cultured in NSC medium were treated with vehicle or 5 μM Akti-1/2 for 5 days. The whole cell lysates were subjected to western blot analysis using the indicated antibodies. GAPDH served as the loading control. (B) U87 cells were treated in the same manner as (A), and the images were captured under an Olympus IX81 microscope with an Olympus IX-TVAD camera at a magnification of ×100. Scale bar, 100 μm. (C) U87 cells were treated in the same manner as (A), and then subjected to the WST-1 assay. Data are presented as means ± SD of three independent experiments.

We previously demonstrated that Oct4-pT235 promotes the self-renewal and survival of ECCs (25). In the present study, we found that, Akti-1/2, a specific inhibitor of Akt, significantly reduced the level of Oct4-pT235 in U87 neurosphere cells while concomitantly decreasing the Akt-pT308 and Akt-pS473 levels (Fig. 4A). Notably, the sizes of Akti-1/2-treated U87 neurospheres were much smaller than those of vehicle-treated neurospheres (Fig. 4B), and the proliferation of Akti-1/2-treated neurosphere cells were markedly attenuated (Fig. 4C).

### ITE decreases the Oct4-pT235 level and inhibits the proliferation of stem-like cancer cells in neurospheres and in a mouse xenograft model

**Figure 5:**
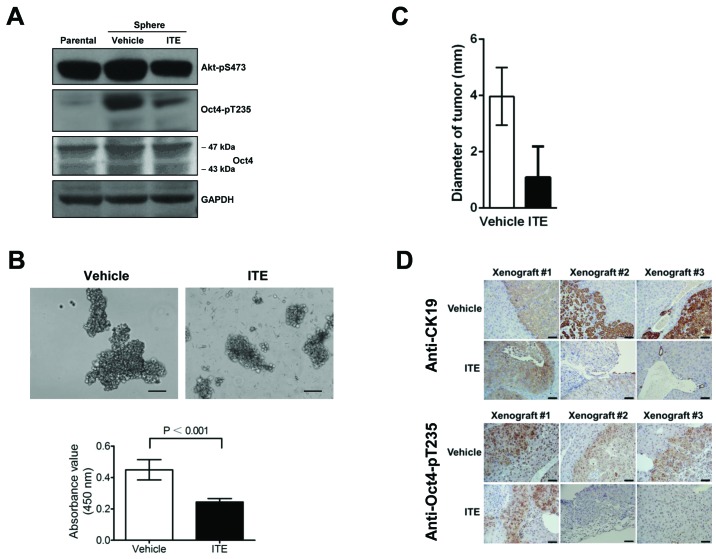
ITE decreases the Oct4-pT235 level and inhibits the proliferation of stem-like cancer cells in neurospheres and in a mouse xenograft model. (A) Adherent parental U87 cells (Parental), U87 neurosphere cells treated with vehicle (Vehicle) or 10 μM ITE (ITE) for 3 days were collected, and analyzed by western blotting for the indicated proteins. (B) U87 neurosphere cells were treated in the same manner as (A), and the images were captured under an Olympus IX81 microscope with an Olympus IX-TVAD camera at a magnification of ×100. Scale bar, 100 μm. the cells were then subjected to the WST-1 assay as described in Materials and methods. Data are presented as means ± SD of three independent experiments. (C) HCCLM3 cells were orthotopically transplanted into the livers of nude mice. After 2 weeks, the mice were intraperitoneally injected with DMSO (Vehicle) or ITE at a dose of 80 mg/kg/day for 15 consecutive days, and were then sacrificed. The excised tumor tissues were fixed and stained with H&E to determine the diameter of the tumors. The averaged tumor diameters of 3 mice in each group were calculated and plotted. (D) The fixed tumor tissues of the 3 mice in each group as described in (C) were stained with anti-CK19 (top panel) or anti-Oct4-pT235 (bottom panel), respectively, and counterstained with hematoxylin. Scale bars, 50 μm.

Emerging evidence indicated an intrinsic connection between the PI3K/Akt signaling pathway and the aryl hydrocarbon receptor (AhR), a ligand-activated transcription factor responding to environmental toxicants. For instance, AhR-deficient hepatoma cells exhibited impaired activation of Akt and enhanced sensitivity to apoptosis (34). β-naphthoflavone (BNF), an agonist of the AhR and a putative chemotherapeutic agent, inhibited PI3K/Akt signaling in breast cancer cells (35). We demonstrated that ITE, another agonist of the AhR that possesses antitumor activities (29), significantly reduced the Oct4-pT235 level in U87 neurospheres (Fig. 5A) and inhibited their proliferation *in vitro* (Fig. 5B). To better mimic the organ and tissue microenvironment in which the tumors grow, we orthotopically transplanted the human hepatocellular HCCLM3 carcinoma cells into the livers of nude mice. Hematoxylin-eosin (H&E) staining showed that all three vehicle-treated transplanted mice bore prominent tumors, while only one out of three ITE-treated mice was found to contain growing tumor, and the average tumor size of the ITE group was smaller than that of the vehicle group (Fig. 5C). Staining for the hepatocellular carcinoma marker cytokeratin-19 (CK19) further confirmed the observations with the H&E staining and revealed typical and expected cytological characteristics of the transplanted HCCLM3 cells (Fig. 5D). In the vehicle group, the levels of Oct4-pT235 in the implanted HCCLM3 cells were significantly higher than those in adjacent mouse liver cells, indicating that this site-specific phosphorylation of Oct4 may be associated with tumorigenesis and/or the maintenance of tumors. By contrast, there were only background levels of Oct4-pT235 in two of the ITE-treated mice (Fig. 5D), further confirming the role of ITE in reducing the Oct4-pT235 level as seen above in U87 neurosphere cells (Fig. 5A).

## Discussion

The presence of Oct4A protein in normal or cancerous adult tissues or cells has been widely questioned due to the lack of convincing data. In this study, using a well-characterized monoclonal anti-Oct4A antibody, we detected two bands (of 43 and 47 kDa) in western blots that can be significantly reduced by an shRNA targeting *POU5F1* and *OCT4-pg1*, indicating they are highly associated with Oct4A protein. Detection of the upper band (47 kDa) by anti-Oct4-pT235 further confirmed its close association with Oct4A. Given the extremely high degree (95%) of sequence homology between Oct4A and Oct4-pg1 proteins, it is difficult to distinguish them at the protein level using routine approaches. Thus, it is critical in future study to purify the endogenous 47 kDa proteins and to fully identify them by amino acid sequencing. More importantly, irrespective of whether the 47 kDa protein corresponds to Oct4A or its pseudogene product, additional investigations are required to fully characterize its target genes and functional roles in the context of human cancer cells.

To the best of our knowledge, we have shown for the first time that a specific site in Oct4A or its homolog protein, which corresponds to T235 in human Oct4A, in human cancer cells can be phosphorylated by serine/threonine-protein kinase Akt. When U87 glioblastoma cells were cultured in NSC medium to induce the formation of neurospheres that are rich in glioblastoma stem-like cells, the level of Oct4-pT235 in sphere cells was markedly increased compared to their monolayer parental cells. Consistent with previous studies (36,37), we found the PI3K/Akt pathway to be generally upregulated in U87 sphere cells. Emerging evidence indicates that PI3K/Akt signaling is critical for the self-renewal of stem-like cancer cells and tumor progression (38,39). Moreover, the aberrantly activated Oct4/Tcl1/Akt signaling pathway contributed to chemotherapeutic drug resistance in liver cancer cells (40). The effects of Akt were thought to be mediated by the activation of the Wnt/β-catenin pathway and GSK3β phosphorylation in mammary stem/progenitor cells (41), and by Akt-induced ABCG2 activation in glioblastoma stem-like cells (42).

The results of the present study suggest that Oct4 may be another important substrate for Akt in maintaining the self-renewal of stem-like cancer cells. It is noteworthy that among the examined human cancer cell lines, U87 exhibited the lowest Oct4 mRNA level but the highest Oct4 protein level and Oct4-pT235 level. Since our previous study has shown that Akt-mediated phosphorylation at the T235 site stabilizes Oct4 protein by attenuating its proteasome-dependent degradation (25), it is possible that a high level of Oct4-pT235 along with other PTMs may facilitate the stabilization of Oct4 protein and increase its steady-state level in U87 cells. In this study, we established that reducing the Oct4-pT235 level by either Akti-1/2 or ITE is associated with attenuated proliferation of stem-like cancer cells *in vitro* and *in vivo*. Future studies are required to assess the potential value of Oct4-pT235 as a diagnostic, prognostic, or predictive biomarker for certain types of human cancer, and to fully decipher the complete Akt-Oct4 interplay system in stem-like cancer cells which may be useful in revealing the intrinsic connection between self-renewal and the tumorigenesis of those cells, and in developing more effective interventions to eradicate or inhibit them.
